# Ultra high-field (7tesla) magnetic resonance spectroscopy in Amyotrophic Lateral Sclerosis

**DOI:** 10.1371/journal.pone.0177680

**Published:** 2017-05-12

**Authors:** Nazem Atassi, Maosheng Xu, Christina Triantafyllou, Boris Keil, Robert Lawson, Paul Cernasov, Elena Ratti, Christopher J. Long, Sabrina Paganoni, Alyssa Murphy, Nouha Salibi, Ravi Seethamraju, Bruce Rosen, Eva-Maria Ratai

**Affiliations:** 1 Neurological Clinical Research Institute (NCRI), Department of Neurology, Massachusetts General Hospital, and Harvard Medical School, Boston, Massachusetts, United States of America; 2 Department of Radiology, First Affiliated Hospital of Zhejiang Chinese Medical University, Hangzhou, P.R. China; 3 Department of Radiology, Massachusetts General Hospital, Athinoula A. Martinos Center for Biomedical Imaging, and Harvard Medical School, Charlestown, Massachusetts, United States of America; 4 Massachusetts Institute of Technology, Sloan School of Management, Cambridge, Massachusetts, United States of America; 5 Department of Physical Medicine and Rehabilitation, Spaulding Rehabilitation Hospital, Charlestown, Massachusetts, United States of America; 6 Siemens Healthineers, MR R&D, Siemens, Auburn, Alabama, United States of America; 7 Siemens Healthineers, MR R&D, Siemens, Charlestown, Massachusetts, United States of America; Linköping University, SWEDEN

## Abstract

The main objective of this study was to utilize high field (7T) *in vivo* proton magnetic resonance imaging to increase the ability to detect metabolite changes in people with ALS, specifically, to quantify levels of glutamine and glutamine separately. The second objective of this study was to correlate metabolic markers with clinical outcomes of disease progression. 13 ALS participants and 12 age-matched healthy controls (HC) underwent 7 Tesla MRI and MRS. Single voxel MR spectra were acquired from the left precentral gyrus using a very short echo time (TE = 5 ms) STEAM sequence. MRS data was quantified using LCModel and correlated to clinical outcome markers. N-acetylaspartate (NAA) and total NAA (tNA, NAA + NAAG) were decreased by 17% in people with ALS compared to HC (P = 0.004 and P = 0.005, respectively) indicating neuronal injury and/or loss in the precentral gyrus. tNA correlated with disease progression as measured by forced vital capacity (FVC) (P = 0.014; R_ρ_ = 0.66) and tNA/tCr correlated with overall functional decline as measured by worsening of the ALS Functional Rating Scale-Revised (ALSFRS-R) (P = 0.004; R_ρ_ = -0.74). These findings underscore the importance of NAA as a reliable biomarker for neuronal injury and disease progression in ALS. Glutamate (Glu) was 15% decreased in people with ALS compared to HC (P = 0.02) while glutamine (Gln) concentrations were similar between the two groups. Furthermore, the decrease in Glu correlated with the decrease in FVC (P = 0.013; R_ρ_ = 0.66), a clinical marker of disease progression. The decrease in Glu is most likely driven by intracellular Glu loss due to neuronal loss and degeneration. Neither choline containing components (Cho), a marker for cell membrane turnover, nor myo-Inositol (mI), a suspected marker for neuroinflammation, showed significant differences between the two groups. However, mI/tNA was correlated with upper motor neuron burden (P = 0.004, R_ρ_ = 0.74), which may reflect a relative increase of activated microglia around motor neurons. In summary, 7T ^1^H MRS is a powerful non-invasive imaging technique to study molecular changes related to neuronal injury and/or loss in people with ALS.

## Introduction

Amyotrophic Lateral Sclerosis (ALS) is a progressive neurodegenerative disorder affecting the upper and lower motor neurons leading to muscle atrophy, weakness, and death due to respiratory failure. To this date, no treatment prevents, halts, or reverses the disease; though the FDA-approved drug riluzole provides a modest survival benefit. The pathogenesis of ALS is not very well understood [1]. Proposed pathogenic mechanisms include mitochondrial dysfunction, glutamate-mediated excitotoxicity, endoplasmic reticulum stress, free radical-mediated oxidative cytotoxicity, and neuroinflammation including microglia activation and astrogliosis [2]. Altogether, these mechanisms lead to progressive neuronal loss. Studies of the SOD1 rodent models of ALS suggest that glutamate excitotoxicity and neuroinflammation are the drivers of neuronal death and ALS progression [3, 4].

Proton magnetic resonance imaging (^1^H MRS) is a neuroimaging technique that allows quantifying metabolic changes *in vivo*. Thus, ^1^H MRS has the potential to provide surrogate markers to assess disease progression and monitor therapeutic treatments in ALS. Specifically, ^1^H MRS is able to quantify metabolites such as N-acetylaspartate (NAA), a marker of neuronal integrity; creatine (Cr), a marker of energy metabolism; choline-containing compounds (Cho), a marker of cell membrane turnover; myo-inositol (mI) a marker of glial activation, and several neurotransmitters and their precursors e.g. γ-aminobutyric acid (GABA), glutamate (Glu), and glutamine (Gln). Indeed, there is a vast body of literature relating to the evaluation of ALS using ^1^H MRS at 1.5 Tesla and 3 Tesla [5–9].

The majority studies have described a reduction in NAA concentrations [10–12] or NAA ratios (e.g. NAA/Cr or NAA/Cho) [13–21] in the motor cortex of people with ALS compared to heathy controls suggesting either neuronal loss or injury in the motor cortex of ALS patients. While measurements of NAA concentrations have provided to be reliable in ALS, measurements of other metabolites and neurotransmitters at 3T have been inconsistent.

Increased Cho and Cho/Cr levels have been reported in people with ALS [15, 22–24] in some studies but not others [16, 21, 25, 26]. Elevated mI and mI/Cr have been detected in the motor cortex in people with ALS [15, 22, 27, 28] but not in early-stage ALS [29].

^1^H MRS reports on Glu and/or the sum of Glu and Gln are also variable. On MR scanners operating at 1.5T or 3.0T, the resonances of Glu and Gln overlap significantly and are commonly referred to as Glx. Early studies found no significant differences in Glx and Glu levels [10, 28] in the motor cortices, occipital cortex, and brain stem, while some other studies reported decreased Glu levels in the precentral gyrus in patients with ALS compared with controls [15]. Using a TE-averaged Point Resolved Spectroscopy (PRESS) sequence at 3T, Han et al. reported that both Glu/Cr and Glx/Cr were increased in the motor cortices as well as in the posterior limb of the internal capsule [30]. Furthermore, early magnetic resonance spectroscopic imaging (MRSI) studies by Pioro et. al reported increased levels Glx in the medulla in people with ALS compared to healthy controls [13, 14].

The objective of this study was to utilize ultra-high field (7T) ^1^H MRS to increase our ability to detect metabolite changes in people with ALS, specifically, to quantify levels of Glu and Gln separately. The second objective of this study was to correlate these metabolic markers with clinical outcomes of disease progression.

## Materials and methods

The study was approved by the Partners Human Research Committee. All participants provided written informed consent.

### Study participants

Fourteen people with ALS according to the revised El Escorial criteria and 12 age-matched healthy controls (HC) were enrolled and underwent 7T MRS. Eligibility criteria included being able to lie flat for at least 45 minutes and considered medically safe to tolerate MRI scanning. Healthy age-matched controls (HC) were recruited from our registry of healthy volunteers.

Mean age did not differ between HC (52±10 years old) and people with ALS (56±10 years old) (P = 0.21). Of the 26 participants, 13 participants with ALS (10 male, 3 female, 56±10 y/o, age range 37–76 years) and 12 healthy controls (6 male, 6 female, 52±10 y/o, age range 37–78 years) had good quality MRS data, and were included in the analysis. Baseline characteristics of the 13 ALS participants are summarized in Table 1.

**Table 1:** Patient characteristics.

	ALS (n = 13)		HC (n = 12)	
	Means	SD	Means	SD
**Age (years)**	56	10	52	10
**Gender (male/female)**	10/3		6/6	
**Disease Duration (months)**	31.2	22.6		
**Time Since Diagnosis (months)**	19.8	18.4		
**ALSFRS-R Score**	38.4	4.7		
**FVC**	93.8	21.3		
**UMNB Score**	22.8	6.5		
**ΔFS**	0.44	0.34		

ALSFRS-R: ALS Functional Rating Scale revised, FVC: forced vital capacity; UMNB: upper motor neuron burden, ΔFS: disease progression, SD: standard deviation

Clinical measures included the worsening of the ALS Functional Rating Scale–revised (ALSFRS-R), forced vital capacity (FVC), and upper motor neuron burden (UMNB). The ALSFRS-R is a quickly administered (10 minutes) ordinal rating scale (ratings 0–4), which determines patients' assessment of their capability and independence in 12 functional activities including bulbar, gross motor scale (GMS), fine motor scale (FMS), and respiratory performance [31]. We estimated the rate of disease progression (ΔFS) using the following formula: (48—ALSFRS-R)/disease duration [32]. FVC is measured using a spirometry in an upright position, and is used to assess diaphragmatic weakness in people with ALS. The UMNB score reflects measurement of the following deep tendon (scores 0–4) and pathological reflexes (present—1 or absent—0): biceps, brachioradialis, triceps, knee jerk, ankle jerk, Hoffman, Babinski, and jaw jerk. The total UMNB score ranges from 0 to 45, with 0 representing areflexia and 45 representing the highest possible UMN burden.

### Data acquisition

All *in vivo* MRI experiments were performed on a 7 Tesla MR imager (Siemens AG Erlangen) using a transmit birdcage coil and a 32-channel receive coil. MRS data was prescribed based on the axial, coronals, and sagittal reformatted images obtained by a multi-echo MPRAGE sequence with 1mm^3^ isotropic resolution, GRAPPA acceleration factor 2, TR = 2530 TE(1) = 1.44 ms, TE(2) = 3.18 ms, TE(3) = 5.04 ms, and TE(4) = 6.9 ms.

**Fig 1:**
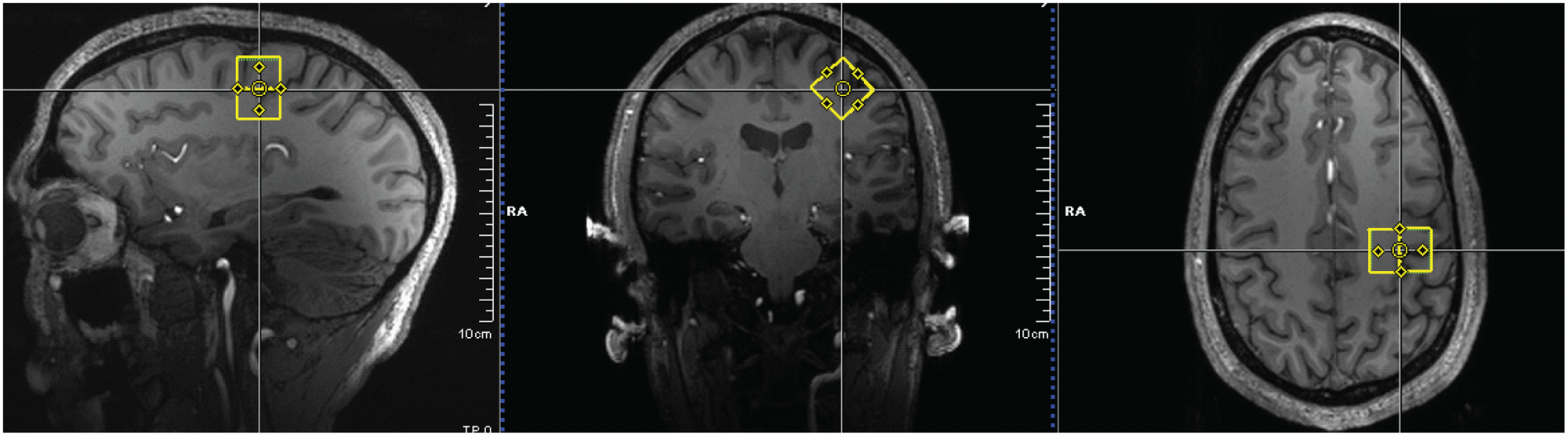
Placement of a 2x2x2cm^3^ voxel on the left precentral gyrus overlaid on a sagittal, coronal and axial reformatted MPRAGE. The voxel does not appear to be square as the voxel is oblique to the sagittal and axial view and the graph shows the voxels’ projections.

Single voxel ^1^H MR spectra were acquired from the motor cortex (voxel of interest (VOI) = 2x2x2cm^3^) using a very short echo time STEAM sequence with VAPOR water suppression technique (STEAM: STimulated Echo Acquisition Mode and VAPOR: Variable Power and Optimized Relaxation Delays) [33, 34]. Consistent voxel placement across subjects was confirmed via neuroanatomical identification of the precentral gyrus by the same neurologist for all scans (Fig 1).

Very short echo times of 5 ms were used to reduce T2 relaxation effects and J-modulation, which improves the quantification of Glu and Gln. High bandwidth slice-selective radiofrequency (RF) pulses were used to minimize chemical shift displacement errors. Other parameters included TR of 5000 ms, TM of 75 ms, bandwidth of 4k, pulse duration of 2.6 ms, and averages of 96. No outer volume saturation was used. Prior to data acquisition, the MRS ROI underwent an automatic shim routine based on gradient double acquisition (GRE-shim) using first and second order shims followed by first order manual shimming. Average full widths at half maximum (FWHM) of the NAA resonance was 11±1Hz. Water unsuppressed spectra from the same voxel were acquired to estimate absolute concentrations in institutional units (I.U.) and to compensate for eddy currents.

### Spectral quantification

Metabolite quantification was performed with LCModel (version 6.3, Copyright S.W. Provencher) for the following 17 metabolites: alanine (Ala), aspartate (Asp), ascorbate (Asc), creatine (Cr), phosphocreatine (PCr), γ-aminobutyric acid (GABA), glutamine (Gln), glutamate (Glu), glutathione (GSH), glycerophosphocholine (GPC), phosphocholine (PCho), myo-inositol (mI), scyllo-inositol (scyllo-Ins), lactate (Lac), N-acetylaspartate (NAA), N-acetylaspartylglutamate (NAAG), and taurine (Tau). We also measured the ratios of tNA/tCr and mI/tNAA as those were reported as meaningful biomarkers in ALS. Specifically, mI/tNA provided an optimal sensitivity and specificity profile [27].

**Fig 2:**
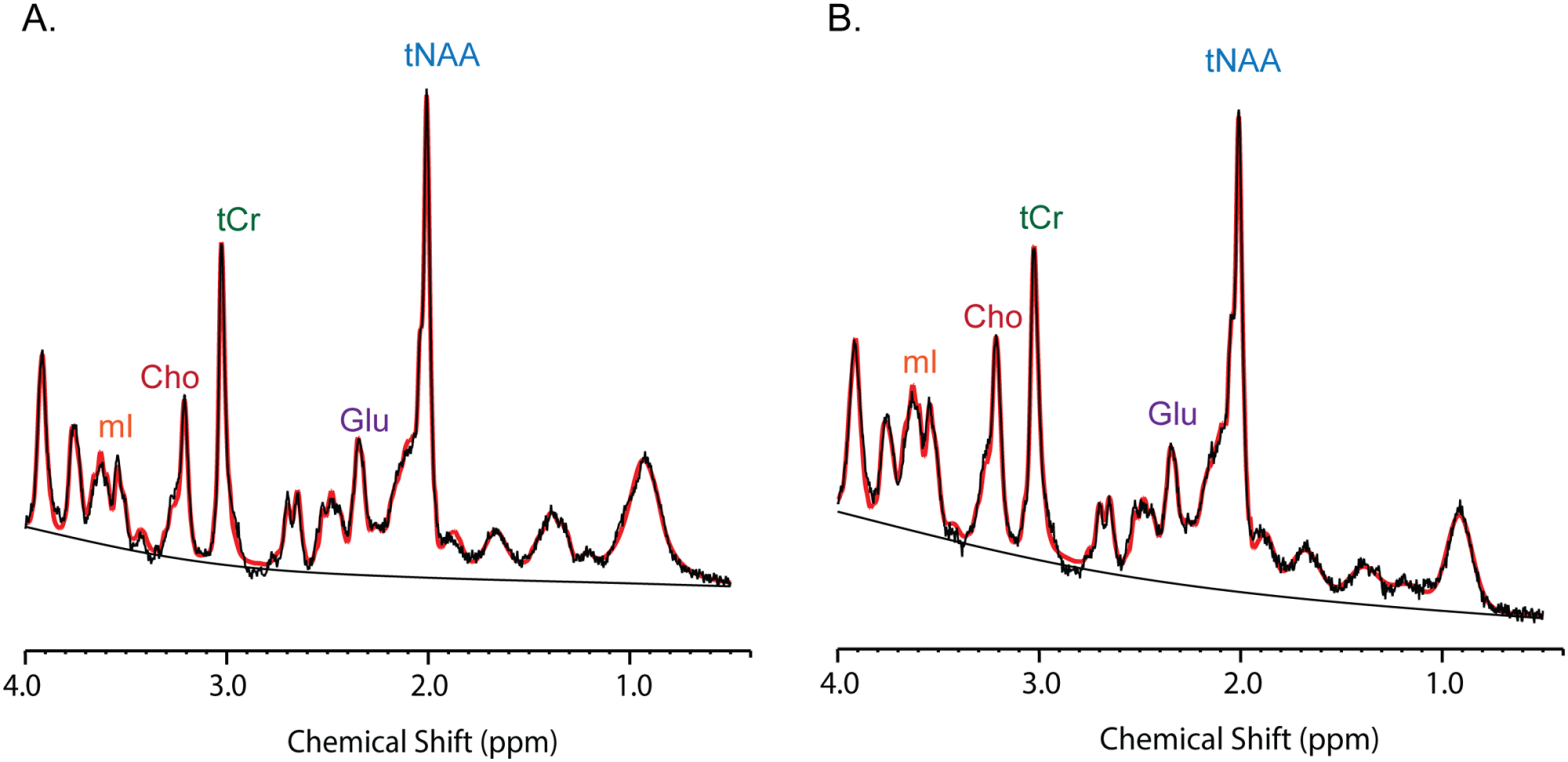
Magnetic resonance spectra representative spectra obtained with very short TE STEAM with VAPOR water suppression (TE 5 ms, TR 5000ms, TM 75 ms, 2 x 2 x 2 cm^3^ voxel size and 96 averages) of a healthy control (A) and an ALS patient (B). Please note the excellent overlay between the acquired data and LCModel fit.

The 7T LC Model basis set was simulated by Dr. Dinesh Deelchand from the Center for Magnetic Resonance Research at the University of Minnesota using Matlab based on density matrix formalism and an updated database of chemical shifts and coupling constants [35, 36], as described previously [37]. Averaged spectra macromolecules using metabolite nulling inversion-recovery experiments (acquisition parameters: TR = 2s and TI = 675ms) were also included in the 7T LCModel basis sets. The LCModel analysis was performed on spectra within the chemical shift range 0–4ppm. The signal to noise ratio (SNR) was estimated in LCModel and is defined as the peak height of NAA divided by the root mean square of the noise of the LCModel fit. The control parameters that LC Model uses for calculation of metabolite concentration from a water reference were not changed from the default values. Two representative spectra of an ALS patient compared to an age-matched control are shown (Fig 2).

The multi echo MPRAGE sequence was used to segment brain volume of white matter, gray matter, or cerebrospinal fluid (CSF) within the 2x2x2cm^3^ MRS voxel using FMRIB Software Library (FSL 5.0, Oxford, UK). All metabolite levels were adjusted for CSF contribution.

### Statistical analysis

The primary focus of this study was 1) to determine the directionality of glutamate in the motor cortex of people with ALS and 2) to evaluate metabolic changes related to neuronal injury (NAA). For these primary hypnotizes, two-tailed Student t-tests were used to compare Glu and NAA concentrations between ALS subjects and healthy controls and multiple testing Bonferroni correction of P = 0.05/2 = 0.025 was made.

Other metabolites (NAAG, Gln, mI, GPC, GSH, tCr and GABA) were examined for further exploratory analyses. Again, two-tailed Student t-tests were used to compare metabolite concentrations between ALS subjects and healthy controls. For these additional exploratory metabolites, no corrections for multiple comparisons were made. All analyses were conducted using JMP 10.0 (SAS, Cary, NC). Spearman correlation coefficients were calculated to estimate the correlations between MRS markers and clinical outcomes, e.g. FVC, UMNB and worsening of the disease progression (ΔFS). For that, only metabolites/ metabolite ratios were selected for this analysis that showed a significant difference or a trend between people with ALS and HC. For the correlational analysis, multiple testing Bonferroni correction of P = 0.05/3 = 0.017 was made.

## Results and discussion

### Subjects

Fourteen people with ALS and 12 age-matched healthy controls (HC) were enrolled and underwent 7T MRS. All HC and ALS participants tolerated the ~45 min scan. However, ALS as well as HC participants reported dizziness when moved within the fringe field of the 7T, which subsided after lying still in the scanner.

### VOI segmentation

Since the single voxel VOI encompassed CSF, gray matter, and white matter, the VOI of each subject was segmented using FSL. VOI included on average 61%±9% white matter, 28%±7% gray mater, and 11%±9% CSF. Of note, there was no significant difference between ALS and HC subjects in the ratios of CSF (P = 0.76), gray matter (P = 0.71) and white mater (P = 0.39). To verify that none of the compartments were either under- or overestimated, we overlaid the MRS voxel with the MNI template using FSL. While we have no gold standard to assess the performance of the FSL segmentation tool, we did manually inspect all of its outputs.

### Metabolites

The SNR estimated in LCModel (defined as the peak height of NAA divided by the root mean square of the noise of the LCModel fit) was 40±7. Analyses are based on 13 ALS patients and 12 HC. One patient with ALS was excluded because he was unable to complete the MRS scan.

**Fig 3:**
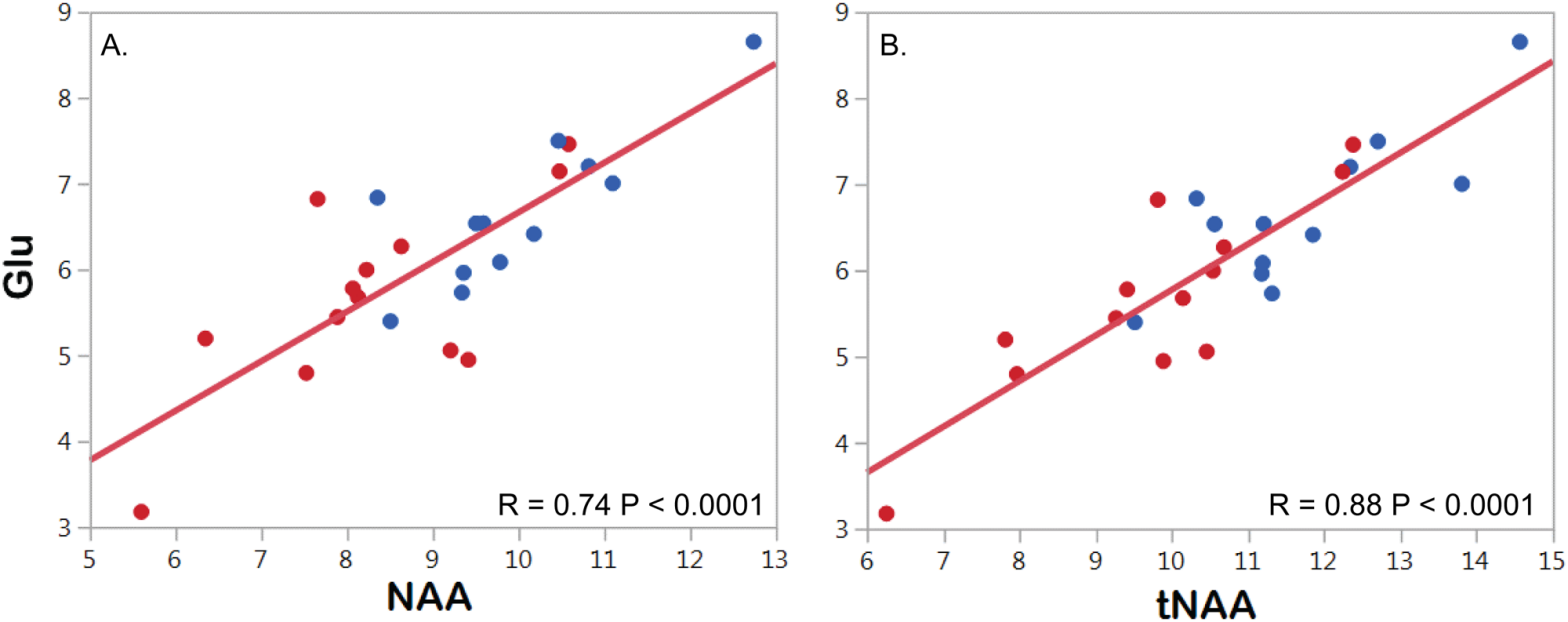
Correlation between NAA and glutamate (Glu) (P<0.0001, R_ρ_ = 0.74) and between total NAA (tNA) and Glu (P<0.0001, R_ρ_ = 0.88), blue circles representing heathy control and red circles representing ALS data.

The comparison between ALS participants and healthy controls revealed a decrease of 17% in NAA in people with ALS compared to healthy controls (P = 0.004) (Table 2) while no changes in NAAG were observed (P = 0.25). Glutamate levels were 15% decreased in people with ALS (P = 0.02) while no changes in Gln were observed (P = 0.99). There was a correlation between NAA and Glu (P<0.0001, R_ρ_ = 0.74, Fig 3), but not between NAA and Gln (P = 0.3, R_ρ_ = 0.18). GABA, GSH, and tCr levels were not significantly different in ALS patients compared to the levels in HC, (P = 0.16, P = 0.14, and P = 0.16, respectively). Neither choline containing components nor mI levels showed significant differences between the two groups.

**Table 2:** Absolute metabolic concentrations (mM in institutional units) and relative metabolic concentrations of the left motor cortex in ALS patients vs. age-matched healthy controls.

Metabolite	ALS (n = 13)		HC (n = 12)		Change %	P Value
	Means	SD	Means	SD		
**NAA**	8.29	1.43	9.98	1.21	**-17%**	**0.004**^#^
**NAAG**	1.48	0.580.63	1.74	0.48	-15**%**	0.25
**NAA+NAAG (tNA)**	9.77	1.70	11.71	1.45	**-17%**	**0.005**
**Gln**	1.73	0.47	1.73	0.46	0.0**%**	0.99
**Glu**	5.67	1.13	6.65	0.88	**-15%**	**0.02**^#^
**mI**	5.71	1.55	5.79	1.40	-1**%**	0.89
**GPC**	1.17	0.33	1.25	0.27	-6**%**	0.53
**GSH**	1.08	0.31	1.25	0.25	-14**%**	0.14
**tCr**	7.09	1.47	7.89	1.29	-10**%**	0.16
**GABA**	0.45	0.35	0.64	0.33	-31**%**	0.16
**tNA/tCr**	1.393	0.149	1.493	0.105	-7**%**	***0*.*06***
**mI/tNA**	0.584	0.121	0.493	0.080	18**%**	**0.03**

^#^ Bonferroni corrected P = 0.025.

Abbreviations: NAA, N-acetylaspartate; NAAG, Nacetylaspartylglutamate; tNA, total NAA; Glu, Glutamate; Gln Glutamine; mI, myo-Inositol; GPC, Glycerophosphocholine; GSH, Glutathione; tCr, total creatine, GABA, gamma-Aminobutyric acid;

We also measured the ratios of tNA/tCr and mI/tNA as those were reported as meaningful biomarkers in ALS. Specifically, mI/tNA provided an optimal sensitivity and specificity profile [27]. tNA/Cr was 7% decreased in people with ALS, however, these differences only showed a trend toward significance (P = 0.06). Finally, people with ALS had 18% higher mI/tNA levels compared to healthy controls (P = 0.03).

In the appendix (S1 Table), we have extended our analysis to all 17 metabolites of the basis-set.

### Correlations between metabolism and clinical measures

Lastly, we investigated if any of the clinical outcome measures in ALS correlated with the metabolic concentrations or metabolic ratios (Table 3). We only selected metabolites/ metabolite ratios that showed a significant difference or a trend between people with ALS and HC. FVC correlated negatively with tNA and Glu. Upper motor neuron burden (UMNB) correlated positively with mI/tNA (P = 0.004, R_ρ_ = 0.74). Lastly, Spearman Rank correlations showed a negative relationship between ΔFS and tNA/tCr (P = 0.028, R_ρ_ = -0.60).

**Table 3:** Correlations between clinical outcomes and metabolites/metabolite ratios in ALS.

MRS marker	Clinical outcome	Spearman R_ρ_	P value
NAA+NAAG (tNA)	FVC	0.66	0.014
Glu	FVC	0.66	0.013
mI/tNA	UMNB	0.74	0.004
tNA/tCr	ΔFS	-0.60	0.028

Bonferroni corrected P = 0.017

UMNB: upper motor neuron burden; ΔFS: disease progression; FVC: forced vital capacity

The development of *in vivo* biomarkers to aid in early diagnosis, to understand disease mechanisms, and to serve as readout of experimental treatments is an unmet need in ALS. Neuroimaging is an emerging candidate as a source of biomarkers for ALS. Here, we used 7 Tesla MRS to quantify brain metabolites and correlate them with ALS disease progression. Previous studies have demonstrated the ability of high field *in vivo* ^1^H MRS to increase detection sensitivity for those compared to lower field strengths [36]. In addition, we utilized an MRS sequence with very short echo times of 5 ms to reduce T2 relaxation effects and J-modulation, which improves the quantification of Glu and Gln.

Our findings of decreased NAA and tNA in ALS are consistent with the findings from previous 1.5T and 3T MRS studies in ALS [10–12, 14–21, 30, 38]. Within the adult brain, NAA is found predominantly in neurons, and serves as a marker of neuronal integrity and mitochondrial dysfunction [39]. Thus, the decrease in NAA can be attributed to either neuronal loss or injury. While most studies described a reduction in tNA concentrations or tNA ratios (tNA/tCr) in the motor cortex of people with ALS, a few recent reports showed that tNA relative to water did not significantly differ between people with ALS and HC, merely the ratio of NAA/Cr decreased [26, 40] suggesting small opposite changes in NAA and tCr in ALS. Our study reveals both tNA and tNA ratios over tCr are decreased in the motor cortex people with ALS. Furthermore, we could not confirm an increase in tCr in our study.

Spearman Rank correlations showed an inverse relationship between rate of disease progression (ΔFS) and tNA/tCr. These results indicate that neuronal injury/loss is more prominent in ALS participant in whom the disease is progressing at a faster rate. Furthermore, a decrease in tNA was associated with a decrease in forced vital capacity, another measure of disease progression. A number of studies have also explored correlations between tNA and clinical scales, and have reported significant associations among tNA levels and ALSFRS-R scores [12, 17, 41], UMNB burden scores [16, 17, 41], and rate of disease progression [42]. Thus, our study confirms the importance of NAA as reliable biomarker for neuronal injury and disease progression.

There is sufficient evidence to support that excitotoxicity may contribute to ALS pathophysiology [3, 4]. Glutamate is the major excitatory neurotransmitter in the CNS and is involved in the pathogenesis of several neurological disorders when its extracellular concentration rises to toxic levels, termed excitotoxicity [43, 44]. The amino acid Gln is a precursor and storage form of Glu, which is predominantly located in astrocytes [45]. Glutamate levels are increased in the serum and CSF of people with ALS [46]. Previous *in vivo* MRS studies in people with ALS used low magnetic fields (1.5 or 3 Tesla), and reported variable results on Glu and Glx [10, 15, 28, 30]. The development of higher magnetic field strengths makes this separation more feasible. Thus, ultra-high magnetic fields such as 7T are desired to accurately quantify glutamate and glutamine separately. Furthermore, we have utilized a STEAM sequence using very short echo times of 5 ms to reduce T2 relaxation effects and J-modulation, which improves the quantification of Glu and Gln.

Our study shows that glutamate levels are decreased in the ALS group compared to healthy controls, whereas glutamine levels are not different. Furthermore, Glu correlated positively with FVC, which is a measure of disease progression.

We hypothesize that the observed decrease in glutamate could be secondary to the loss of intracellular glutamate due to neuronal loss, as reflected in the decreased NAA levels. MRS cannot identify whether the source of metabolic concentrations is from intra- or extra-cellular spaces. Glutamate is predominantly an intracellular metabolite and its role in excitotoxicity probably stems from increases in its extracellular concentrations. However, Glu levels in CSF and in extracellular compartment are at least by three orders of magnitude lower than those in intracellular compartment [47], thus glutamate concentrations measured by MRS represent mostly the intracellular glutamate concentrations, and the reduction of glutamate we observed in the ALS group is probably secondary to intracellular glutamate depletion due to neuronal loss. This hypothesis is supported by the significant positive correlation between Glu and NAA. Glutamate predominantly shares the same compartment as NAA—namely neurons [48–50]. Therefore, a strong correlation between Glu and NAA (Fig 3) could indicate neuronal loss. Our results are consistent with reported decrease in Glu or Glx in other neurodegenerative diseases including Parkinson’s disease [51] and Alzheimer’s disease [52].

In our study, none of the glial markers mI, GPC or tCho was significantly elevated in ALS patients compared to controls. However, we saw a positive correlation between the markers of glial proliferation/inflammation mI/NAA and clinical measures of upper motor neuron dysfunction (increased reflexes) in the ALS group. These findings are in line with ALS *postmortem* pathological studies showing activated microglia around motor neurons [53–57]. Furthermore, a recent PET study revealed increased [^11^C]-PBR28 binding in the precentral gyrus in patients with ALS compared to controls reflecting increased glial activation [58].

This study has a few limitations that we like to discuss below:

1. For the LCModel analysis. we did not change the default control parameters that LC Model uses for calculation of metabolite concentration from a water reference, which may explain why the reported metabolite concentrations are on the lower side. LC Model assumes a default concentration for water of 0.3588 assuming 100% white matter and a signal attenuation factor of 0.7 to account for T2 relaxation assuming a TE of ~ 30 ms. On the other hand, applying a different scaling factor to both groups will not change the statistics between people with ALS and heathy controls or the correlations between the metabolites and the clinical outcome markers.
2. Of note, virtually all metabolite levels were decreased in ALS patients. To ensure, we simple did not simple measure atrophy in the motor cortex of ALS patients, we calculated CSF contribution of each voxel using FSL and corrected the metabolite concentrations for it. An alternative approach to eliminate CSF contributions as potential confounding factor is to report metabolic ratios, typically over total creatine. However, reporting ratios of creatine may be problematic as one cannot be certain that creatine is stable in ALS patients.
3. One of the major limitations include the relatively small sample size and multiple testing which may result in type 1 error. The primary focus of our study was to evaluate metabolic changes related to neuronal injury (NAA) and to determine the directionality of Glu in the motor cortex of people with ALS; for these primary hypnotizes, multiple testing Bonferroni correction of P = 0.05/2 = 0.025 was made. Other metabolites were examined for further exploratory analyses.

For the correlation analyses, we limited tests between the clinical markers and MRS markers to only the metabolites/metabolite ratios that showed a significant difference or a trend between people with ALS and HC. Furthermore, multiple testing Bonferroni correction of P = 0.05/3 = 0.017 was made. We consider this work an important pilot and hypothesis-generating study that will require future confirmation and we plan to use strict correction for multiple testing in future larger study.

## Conclusion

In conclusion, ultra-high field MRS is a powerful and non-invasive imaging technique to study molecular changes of neuronal injury in people with ALS. Future studies that include longitudinal imaging in people with ALS will allow to study metabolite changes as disease progresses, and will help clarify the role of ultra-high field MRS as a biomarker of target engagement for proof-of-mechanism clinical trials.

## Supporting information

S1 Table Absolute metabolic concentrations (mM in institutional units) of all 17 metabolites analyzed of the left motor cortex in ALS patients (N = 13) vs. age-matched healthy controls (N = 12). (DOCX)
