# Supplementary material for: Ultra high-field (7tesla) magnetic resonance spectroscopy in Amyotrophic Lateral Sclerosis
Source: PLoS One. 2017 May 12;12(5):e0177680. doi: 10.1371/journal.pone.0177680 (PMC5428977; doi:10.1371/journal.pone.0177680)
Supplement: S1 Table — (DOCX) [file pone.0177680.s001.docx]

**S1 Table**

**Absolute metabolic concentrations (mM in institutional units) of all 17 metabolites analyzed of the left motor cortex in ALS patients (N = 13) vs. age-matched healthy controls (N = 12)**

| **Metabolite** |  | **ALS** | |  | **HC** | | **Change** | **P Value** |
| --- | --- | --- | --- | --- | --- | --- | --- | --- |
|  |  | **Means** | **SD** |  | **Means** | **SD** |  |  |
| **NAA** |  | 8.29 | 1.43 |  | 9.98 | 1.21 | **-17%** | **0.004** |
| **NAAG** |  | 1.48 | 0.63 |  | 1.74 | 0.48 | -15% | 0.41 |
| **NAA+NAAG (tNA)** |  | 9.77 | 1.70 |  | 11.71 | 1.45 | **-17%** | **0.005** |
| **Gln** |  | 1.73 | 0.47 |  | 1.73 | 0.46 | 0.0% | 0.99 |
| **Glu** |  | 5.67 | 1.13 |  | 6.65 | 0.88 | **-15%** | **0.02** |
| **mI** |  | 5.71 | 1.55 |  | 5.79 | 1.40 | -1% | 0.89 |
| **GPC** |  | 1.17 | 0.33 |  | 1.25 | 0.27 | -6% | 0.53 |
| **PCho** |  | 0.28 | 0.11 |  | 0.27 | 0.13 | 2% | 0.91 |
| **GSH** |  | 1.08 | 0.31 |  | 1.25 | 0.25 | -14% | 0.14 |
| **PCr** |  | 5.05 | 1.51 |  | 5.40 | 0.94 | -6% | 0.51 |
| **Cr** |  | 1.99 | 0.93 |  | 2.50 | 0.72 | -20% | 0.15 |
| **tCr** |  | 7.09 | 1.47 |  | 7.89 | 1.29 | -10% | 0.16 |
| **GABA** |  | 0.45 | 0.35 |  | 0.64 | 0.33 | -31% | 0.16 |
| **scyllo-Ins** |  | 0.16 | 0.16 |  | 0.12 | 0.11 | 39% | 0.44 |
| **Tau** |  | 1.15 | 0.36 |  | 1.33 | 0.45 | -14% | 0.28 |
| **Ala** |  | 0.15 | 0.20 |  | 0.14 | 0.19 | 7% | 0.91 |
| **Asp** |  | 0.82 | 0.85 |  | 0.34 | 0.44 | 140% | 0.10 |
| **Asc** |  | 0.36 | 0.62 |  | 0.57 | 0.75 | -37% | 0.45 |
| **Lac** |  | 0.54 | 0.42 |  | 0.43 | 0.50 | 24% | 0.58 |

Abbreviations: NAA, N-acetylaspartate; NAAG, Nacetylaspartylglutamate; tNA, total NAA; Glu, Glutamate; Gln Glutamine; GABA, gamma-Aminobutyric acid; mI, myo-Inositol; GPC Glycerophosphocholine; PCho, Phosphocholine; GSH, Glutathione; PCr Phoshocreatine; Cr, Creatine; scyllo-Ins (scyllo-inositol); Tau, Taurine; Ala, Alanine; Asp, Aspartate; Asc, Ascorbate; Lac, Lactate.
